# Retrospective Analysis of the Association Between Sarcopenia and Fall Risk in Older Breast Cancer Patients Using Real World Data (TriNetX)

**DOI:** 10.1002/cam4.71404

**Published:** 2025-11-20

**Authors:** Asmaa Namoos, Rana Ramadan, Dina Ramadan, Annie Liang, Nicholas Thomson

**Affiliations:** ^1^ Virginia Commonwealth University School of Medicine Richmond Virginia USA; ^2^ University of Colorado Anschutz Medical Campus Aurora Colorado USA; ^3^ Alexandria University Alexandria Egypt

**Keywords:** breast cancer, cancer aging, falls, older adults, real‐world data, sarcopenia, survivorship, TriNetX

## Abstract

**Background:**

Falls are a major source of morbidity in older adults and may be particularly consequential for breast cancer survivors, who face vulnerabilities from aging, treatment side effects, and comorbidities. Sarcopenia, the progressive loss of skeletal muscle mass and strength, may exacerbate fall risk but is rarely incorporated into oncology‐focused fall risk assessments.

**Objective:**

To evaluate the association between sarcopenia and the risk of first and repeated falls in older breast cancer patients using real‐world electronic health record data.

**Methods:**

We conducted a retrospective cohort study using the TriNetX platform, including women aged 60–89 years diagnosed with breast cancer between 2010 and 2024. Sarcopenia was identified using ICD‐10 code M62.84. Outcomes included first fall, repeated falls, time to fall, and fall frequency. Analyses included unadjusted comparisons, propensity score matching, and multivariable Cox regression adjusting for demographics, comorbidities, treatments, and prior falls.

**Results:**

Among 884,123 patients (768 with sarcopenia, 883,355 without), crude fall rates were markedly higher in those with sarcopenia (first fall: 24% vs. 2%; repeated falls: 13% vs. < 1%; *p* < 0.0001). Kaplan–Meier analyses showed an eightfold higher risk of first fall and a fourteenfold higher risk of repeated falls. After propensity score matching, fall rates were nearly identical. In fully adjusted Cox models, sarcopenia remained a significant predictor of first fall (HR 1.93, 95% CI 1.61–2.31). Other independent predictors included older age, hypertension, diabetes, obesity, opioid use, and prior falls.

**Conclusion:**

Sarcopenia is a strong crude marker of fall risk in older breast cancer survivors, but much of the excess risk reflects comorbidities. Incorporating sarcopenia screening with comorbidity management may enhance fall prevention in survivorship care.

## 1 Background

Beyond simple accidents, falls in older adults often represent, underlying subtle declines in strength, balance, and cognition, as well as the cumulative toll of chronic illnesses [1, 2, 3]. For breast cancer survivors, these subtle declines are rarely isolated. Instead, they intersect with the physical and psychological aftershocks of cancer treatment, changes in body composition, and the complexities of ongoing medical care [1, 2, 3]. For breast cancer survivors, these risks are rarely isolated. Instead, they intersect with the physical and psychological aftershocks of cancer treatment, changes in body composition, and the complexities of ongoing medical care [4, 5].

Within this context, sarcopenia, defined as the progressive loss of skeletal muscle mass and strength with advancing age, emerges as a silent yet powerful driver of health outcomes [6, 7]. The presence of sarcopenia may not be immediately obvious to clinicians or patients, yet it has profound implications for mobility, resilience, and ultimately, a woman's ability to maintain independence [8]. Among breast cancer survivors, especially older women, sarcopenia is both more common and more clinically relevant, given the additional strain of treatments such as chemotherapy, endocrine therapy, and the burden of comorbidities [9, 10].

Sarcopenia often coexists with frailty and other geriatric syndromes, compounding the risks of disability, falls, and hospitalization in older adult patients [11]. Treatment‐related toxicities, cancer‐associated inflammation, and periods of immobility further accelerate muscle loss in this population [12, 13]. Chemotherapy‐induced peripheral neuropathy, chronic pain, and fatigue may compound deficits in balance and function, rendering breast cancer survivors particularly vulnerable to falls and their downstream consequences [14]. Falls, in turn, can precipitate a cascade of adverse outcomes including fractures, fear of future falls, loss of autonomy, and increased mortality [3].

Despite the recognized impact of sarcopenia on overall health, its role as a risk factor for falls in older women with breast cancer has not been well defined [10]. Existing fall risk models in oncology rarely incorporate direct measures of muscle mass or function, instead relying primarily on demographic and clinical factors. This omission leaves an important gap in both research and practice. Additionally, most large epidemiologic studies have examined the general population, while breast cancer survivors face a unique constellation of risk factors including neuropathy, hormonal changes, cardiovascular and metabolic comorbidities, and the physical consequences of surgery or radiation [15]. The interaction of these factors with sarcopenia remains underexplored.

Breast cancer survivors represent a unique population in whom the interplay between sarcopenia and fall risk is particularly pronounced. Several mechanisms distinguish this group from other cancer populations. Endocrine therapies, such as aromatase inhibitors, accelerate muscle and bone loss by inducing estrogen deprivation [16], while chemotherapy‐induced peripheral neuropathy impairs proprioception and balance [14, 17]. These effects, combined with age‐related hormonal changes, chronic pain, fatigue, and treatment‐related deconditioning, compound the risk of functional decline [4, 5]. In addition, breast cancer is the most prevalent cancer among older women, creating a large survivorship population in whom these intersecting risks are especially relevant [9, 10]. As such, breast cancer provides a clinically important lens through which to examine sarcopenia‐associated fall risk in aging cancer survivors.

Our study addresses this gap by leveraging real‐world data from a large, multi‐institutional health records network to quantify the association between sarcopenia and falls among older women with breast cancer. Specifically, we evaluated the risk of both first and repeated falls in patients with and without sarcopenia, while adjusting for demographic, clinical, and treatment‐related factors. These findings aim to provide robust estimates that can inform survivorship care planning and guide strategies to preserve safety, independence, and quality of life in this vulnerable population.

## 2 Methods

### 2.1 Study Design and Data Source

We conducted a retrospective cohort study using TriNetX, LCC platform, a federated electronic health records research platform that aggregates deidentified clinical data from several large healthcare organizations (HCOs) across the United States [18]. The TriNetX database includes comprehensive longitudinal information on demographics, diagnoses, procedures, medications, and laboratory measurements. Data for this analysis were extracted in July 2025 from the Research Network, which involved 155,911,921 patients from 105 HCOs at the time of extraction. Because the platform's patient data and HCOs are de‐identified in accordance with Section §164.514 of the HIPAA Privacy Rule, *ethical approval was not required*. The study complied with the ethical and reporting standards described by the TriNetX platform under “Publication Guidelines” [19].

### 2.2 Rationale for Inclusion Criteria and Cohort Definition

The study population included women aged 60 through 89 years diagnosed with breast cancer between January 2010 and December 2024. This age range was chosen to focus on older survivors at heightened risk of sarcopenia and falls, while excluding patients above 89 years to maintain HIPAA confidentiality. The study period ensured contemporary treatment practices and consistent ICD‐10 coding. Eligible patients were identified by a confirmed ICD‐10 code for malignant neoplasm of the breast (C50), with at least 1 year of prior medical records to capture baseline comorbidities, medications, and health status. Complete demographic information was required to enable the analysis of sociodemographic disparities and ensure the validity of multivariable models.

Exposure was defined by the presence or absence of sarcopenia, captured using ICD‐10 code M62.84. While this approach allows standardized identification across healthcare systems, it has important limitations.

### 2.3 Outcomes and Covariates

The primary outcome was the occurrence of falls, ascertained using ICD‐10 codes for unspecified fall (W19) and repeated falls (R29.6) after the index date. The index date is defined as the day the patient fulfilled all the eligibility criteria. Secondary outcomes included the frequency of fall events and time to first fall. Covariates included age at index date, race, ethnicity, hypertension, diabetes, obesity, prior falls, cancer treatments (chemotherapy, hormonal therapy, radiation), opioid and antihypertensive use. Cancer stage was available in the dataset but was not included in primary analyses due to incomplete data capture and lack of relevance to the study objective. Fall diagnoses (W19, R29.6) were captured from all available clinical settings, including inpatient admissions, outpatient visits, and emergency department encounters within the TriNetX network.

### 2.4 Propensity Score Matching

To address confounding and simulate randomization, we used one‐to‐one propensity score matching (PSM) between the patients with and without sarcopenia. Propensity scores were calculated using logistic regression based on key demographic and clinical covariates listed above. Nearest‐neighbor matching was performed with a caliper of 0.1 pooled standard deviation, and unmatched patients were excluded. Post‐matching balance was assessed by calculating standardized mean differences for all variables, considering a value below 0.1 as well balanced. The Cox regression was performed on the full analytic sample to preserve statistical power and enable time‐to‐event modeling. The PSM cohort was used as a secondary, exploratory analysis of crude risk comparisons between well‐balanced groups.

### 2.5 Statistical Analysis

Descriptive statistics were used to summarize baseline characteristics. Means with standard deviations and medians were reported for continuous variables; categorical variables were summarized as counts and percentages. Group comparisons were conducted using independent sample *t*‐tests for continuous variables and chi‐square tests for categorical variables. Following matching, primary and secondary outcomes were compared between cohorts using relative risks, absolute risk differences, and 95% confidence intervals. Statistical significance was set at a two‐sided *p* value less than 0.05. Time‐to‐event outcomes were evaluated with Kaplan–Meier survival curves and compared by log‐rank tests. Multivariable Cox proportional hazards regression was used to estimate adjusted hazard ratios for the association between sarcopenia and fall risk, controlling for baseline covariates. All data management and statistical analyses were performed using TriNetX analytic tools. As a secondary analysis, we constructed a multivariable logistic regression model to explore additional covariates available in the dataset that were not included in the primary Cox regression, such as baseline radiology procedures and medication classes (e.g., alpha‐blockers). These were considered exploratory predictors given prior literature linking them to fall risk, but they were not part of our predefined covariate set for the main time‐to‐event models.

## 3 Results

### 3.1 Study Population and Baseline Characteristics

Baseline characteristics of older breast cancer patients by sarcopenia status are presented in Table 1. Patients with sarcopenia were significantly older than those without, with a mean age of 79.5 years (SD 8.93) compared to 74.7 years (SD 9.02; *p* < 0.0001, standardized difference 0.54). At index, the mean age was 76.7 years (SD 9.5) for patients with sarcopenia and 67.0 years (SD 10.1) for those without sarcopenia (*p* < 0.0001, standardized difference 0.99). The age range for both groups was 60–89 years.

**TABLE 1:** Baseline characteristics of older breast cancer patients by sarcopenia status.

Variable	Category	With sarcopenia (*N* = 768)	Without sarcopenia (*N* = 883,355)	*p*	Standard difference
Age	Mean (SD)	79.5 (8.93)	74.7 (9.02)	< 0.0001	0.54
	Range	60 to 90	60 to 90	—	—
Age at index	Mean (SD)	76.7 (9.5)	67.0 (10.1)	< 0.0001	0.99
Race	White	555 (72.3%)	591,231 (66.9%)	0.002	0.12
	Black or African American	130 (16.9%)	87,063 (9.9%)	< 0.0001	0.21
	Asian	29 (3.8%)	39,966 (4.5%)	0.32	0.04
	Other	26 (3.4%)	47,571 (5.4%)	0.014	0.10
	Unknown	20 (2.6%)	109,732 (12.4%)	< 0.0001	0.38
	American Indian or Alaska Native	10 (1.3%)	1879 (0.2%)	< 0.0001	0.13
	Native Hawaiian or Pacific Islander	10 (1.3%)	5913 (0.7%)	0.032	0.06

Racial distributions also differed between groups. White patients comprised 72.3% of the sarcopenia group and 66.9% of the non‐sarcopenia group (*p* = 0.002, standardized difference 0.12). Black or African American patients represented 16.9% versus 9.9%, respectively (*p* < 0.0001, standardized difference 0.21). The sarcopenia group had a lower proportion of patients with unknown race (2.6% vs. 12.4%, *p* < 0.0001, standardized difference 0.38). Smaller but statistically significant differences were observed for American Indian or Alaska Native (1.3% vs. 0.2%, *p* < 0.0001) and Native Hawaiian or Pacific Islander patients (1.3% vs. 0.7%, *p* = 0.032). The proportions of Asian and Other race patients were similar between groups.

### 3.2 Unadjusted Fall Outcomes (Full Cohort)

In the full cohort, the incidence of both first and repeated falls was substantially higher in patients with sarcopenia compared to those without. Among the 768 patients with sarcopenia, 188 (24%) experienced a first fall, whereas 17,487 of 883,355 patients without sarcopenia (2%) experienced a first fall. For repeated falls, 101 sarcopenia patients (13%) reported more than one fall event compared to 3937 patients without sarcopenia (less than 1%) in the much larger comparator group.

Both differences were highly statistically significant (*p* < 0.0001) and reflected large, standardized differences, indicating a strong crude association between sarcopenia and fall risk.

### 3.3 Propensity Score Matched Analysis

Propensity score matching yielded 768 patients with sarcopenia matched one‐to‐one with 768 patients without sarcopenia. Matching achieved excellent balance across demographic and clinical characteristics, with all standardized differences below 0.02, indicating negligible residual imbalance. There were no significant differences between groups in current age (79.5 vs. 79.6 years, *p* = 0.859), age at index (76.7 vs. 76.8 years, *p* = 0.842), or racial composition, including White (72.3 vs. 72.9%), Black or African American (16.9 vs. 16.5%), Asian (3.8 vs. 4.2%), and Other race (3.4 vs. 3.1%) (all *p* > 0.05) (Table 2).

**TABLE 2:** Baseline characteristics and fall outcomes after propensity score matching.

Variables/Outcome	Characteristic	With sarcopenia (*N* = 768)	Without sarcopenia (*N* = 768)	*p*	Standard difference
Age	Current age, mean (SD)	79.5 (8.93)	79.6 (8.91)	0.859	0.009
	Age at index, mean (SD)	76.7 (9.5)	76.8 (9.47)	0.842	0.010
Race	White, *n* (%)	555 (72.3)	560 (72.9)	0.775	0.015
	Black or African American, *n* (%)	130 (16.9)	127 (16.5)	0.838	0.011
	Asian, *n* (%)	29 (3.8)	32 (4.2)	0.695	0.020
	Other race, *n* (%)	26 (3.4)	24 (3.1)	0.774	0.015
Outcome	First fall, *n* (%)	188 (24.5)	189 (24.6)	0.953	0.003
	Repeated fall, *n* (%)	101 (13.2)	95 (12.4)	0.646	0.023

In the matched cohorts, fall outcomes were nearly identical. First falls occurred in 188 of 768 sarcopenia patients (24.5%) and 189 of 768 non‐sarcopenia patients (24.6%, *p* = 0.953, standardized difference 0.003). Repeated falls were reported by 101 patients (13.2%) with sarcopenia and 95 patients (12.4%) without sarcopenia (*p* = 0.646, standardized difference 0.023). These findings suggest that, after balancing for measured covariates, sarcopenia was not independently associated with increased risk of either first or repeated falls.

### 3.4 Unadjusted Fall Outcomes (Analytic Subset)

In the analytic subset used for risk estimation, sarcopenia was strongly associated with increased fall risk prior to adjustment for confounders. Among patients with sarcopenia, 136 of 768 (17.7%) experienced a first fall, compared with 39,426 of 883,355 (4.5%) patients without sarcopenia. This difference corresponded to a risk difference of 13.2 percentage points (95% CI 10.5–15.9), a risk ratio of 4.0 (95% CI 3.4–4.6), and an odds ratio of 4.6 (95% CI 3.8–5.5), all highly significant (*p* < 0.0001).

A similar pattern was observed for repeated falls. In the Sarcopenia group, 75 patients (9.8%) experienced multiple falls compared with 12,179 patients (1.4%) in the non‐Sarcopenia group. This produced a risk difference of 8.4 percentage points (95% CI 6.3–10.5), a risk ratio of 7.1 (95% CI 5.7–8.8), and an odds ratio of 7.7 (95% CI 6.1–9.8), all with *p* < 0.0001.

### 3.5 Time‐To‐Event Analysis (Kaplan–Meier Curves)

Kaplan–Meier analyses demonstrated striking crude differences in fall‐free survival, with a hazard ratio of 8.45 (95% CI 7.14–10.00) before adjustment. In contrast, after adjustment for age, comorbidities, treatment exposures, and prior falls in the Cox regression, the association was substantially attenuated (HR 1.93, 95% CI 1.61–2.31), reflecting the influence of confounding. Crude hazard ratios were obtained from a univariate Cox model paired with Kaplan Meier curves, and group differences were tested with the log rank test.

A similar pattern emerged for repeated falls. The probability of remaining free from multiple falls was 81.8% in patients with sarcopenia compared with 97.9% in those without. The hazard ratio for repeated falls was 14.0 (95% CI 11.2–17.6), again demonstrating a markedly elevated risk among sarcopenia patients (*p* < 0.0001 by log‐rank test). These findings, summarized in Table 3, underscore the significant time‐dependent association between sarcopenia and fall risk.

**TABLE 3:** Kaplan–Meier survival analysis for first and repeated falls (hazard ratios from crude/univariate Cox models; log‐rank *p* values shown for curve comparison).

Outcome	Cohort	Survival probability at end (%)	Hazard ratio (95% CI)	Log‐Rank *p*
First fall	Sarcopenia+	60.3	8.45 (7.14, 10.00)	< 0.0001
	Sarcopenia−	93.2		
Repeated falls	Sarcopenia+	81.8	14.0 (11.2, 17.6)	< 0.0001
	Sarcopenia−	97.9		

### 3.6 Number of Fall Events per Patient

Among patients who experienced at least one fall, the burden of recurrent events was greater in those with sarcopenia. The mean number of fall events was 2.44 (SD 2.77) in the sarcopenia group compared with 1.85 (SD 2.53) in the non‐sarcopenia group. The median number of events was one in both groups, but the difference in mean counts was statistically significant (*p* = 0.006). These results highlight that beyond the risk of falling, sarcopenia was also associated with more frequent fall events.

### 3.7 Adjusted Risk: Multivariable Cox Regression

In the fully adjusted Cox proportional hazards model, sarcopenia remained a significant independent predictor of first fall. Patients with sarcopenia had a 93% higher hazard of experiencing a first fall compared with those without sarcopenia (HR 1.93, 95% CI 1.61–2.31, *p* < 0.0001).

Several other factors were also strongly associated with increased fall risk. Older age at diagnosis, hypertensive disease (HR 2.01), diabetes mellitus (HR 1.46), obesity (HR 1.42), use of antihypertensive (HR 1.44) or opioid medications (HR 2.27), and a prior history of falls (HR 2.91) all significantly elevated risk. Proxies for metastatic disease, including secondary neoplasms involving respiratory, digestive, or other sites, were also independent predictors of fall risk.

By contrast, Black or African American race was associated with a modest reduction in fall risk (HR 0.94, 95% CI 0.91–0.97, *p* = 0.0001). Cancer treatments showed mixed results: chemotherapy (HR 0.98) and radiation therapy (HR 1.34, *p* = 0.0854) were not significantly associated with falls, whereas hormonal contraceptive use was linked to a modest increase in risk (HR 1.24). Detailed estimates from the adjusted Cox model are provided in Table 4.

**TABLE 4:** Adjusted Cox model for first fall (multivariable model controlling for age, comorbidities, medications, and prior falls).

Covariate	Hazard ratio	95% confidence interval	*p*
Sarcopenia (yes vs. no)	1.93	1.61, 2.31	< 0.0001
Age at index (per year)	1.06	1.06, 1.07	< 0.0001
Black or African American	0.94	0.91, 0.97	0.0001
Hypertensive diseases	2.01	1.96, 2.06	< 0.0001
Diabetes mellitus	1.46	1.42, 1.50	< 0.0001
Obesity	1.42	1.37, 1.48	< 0.0001
Antihypertensive use	1.44	1.39, 1.50	< 0.0001
Opioid use	2.27	1.63, 3.16	< 0.0001
History of falling	2.91	2.77, 3.04	< 0.0001
Chemotherapy	0.98	0.95, 1.02	0.3270
Hormonal therapy	1.24	1.14, 1.35	< 0.0001
Radiation therapy	1.34	0.96, 1.88	0.0854
Secondary neoplasm (lymph nodes)	1.04	0.91, 1.18	0.569
Secondary neoplasm (resp/digest)	1.32	1.17, 1.49	< 0.0001
Secondary neoplasm (other)	1.43	1.30, 1.57	< 0.0001
Malignant neoplasm (unspecified)	1.07	1.00, 1.15	0.063

### 3.8 Multivariable Logistic Regression for First Fall

As a secondary analysis, we constructed a multivariable logistic regression model to further examine predictors of first falls. Results were broadly consistent with the Cox model. Increasing current age was independently associated with higher odds of falling (AOR 1.12, 95% CI 1.02–1.23, *p* = 0.022). Clinical exposures, including radiology procedures within the baseline period (AOR 2.05, 95% CI 1.01–4.14, *p* = 0.046) and use of alpha blockers or related medications (AOR 2.92, 95% CI 1.33–6.43, *p* = 0.008), were also significant predictors. Most demographic variables and other comorbidities were not associated with fall risk in this model.

## 4 Discussion

This large retrospective cohort study provides new evidence on the relationship between sarcopenia and fall risk among older women with breast cancer. Using a real‐world, multi‐institutional electronic health record network, we found that sarcopenia was strongly associated with both first and repeated falls in unadjusted analyses. The risk estimates were substantial across multiple statistical approaches, including crude incidence rates, risk ratios, odds ratios, and time‐to‐event analyses. However, once we accounted for demographic, clinical, and treatment‐related differences through propensity score matching and multivariable modeling, the independent effect of sarcopenia was diminished. This finding underscores the complex interplay between sarcopenia and a range of comorbid conditions that collectively shape fall vulnerability in this population.

An important observation is the difference between the crude and adjusted estimates. In unadjusted analyses, women with sarcopenia had dramatically higher fall rates than those without: the proportion experiencing at least one fall was nearly fivefold greater, and the risk of repeated falls was more than sevenfold higher. Kaplan–Meier analysis similarly showed an eightfold higher risk of first falls among sarcopenic patients. Such magnitude of effect aligns with prior work in the general geriatric population, where sarcopenia has consistently emerged as a major predictor of functional decline, hospitalization, disability, and mortality. Systematic reviews and meta‐analyses have highlighted sarcopenia as a potent risk factor for falls, with pooled estimates showing approximately two‐ to threefold higher odds of falling among sarcopenic adults compared with their non‐sarcopenic peers [20]. However, once demographic, clinical, and treatment‐related differences were accounted for through propensity score matching and multivariable modeling, the independent effect of sarcopenia was substantially attenuated, with the Cox regression showing a more modest twofold increase in risk. This attenuation reflects the influence of confounding, as patients coded with sarcopenia were significantly older and had higher burdens of comorbidities such as hypertension, diabetes, and obesity, all of which independently elevate fall risk. Taken together, these findings suggest that sarcopenia is less a stand‐alone cause of falls and more a clinical marker that clusters with other vulnerabilities in older breast cancer survivors [21, 22]. The observed difference between the PSM and Cox model results is expected given that the former reflects unadjusted comparisons between matched groups, while the latter incorporates multivariable adjustment over time. The matched analysis served as a complementary check on crude associations rather than a basis for time‐dependent modeling.

The logistic regression analysis identified radiology procedures and alpha‐blocker use as additional predictors of falls. These findings should be interpreted cautiously, as they were derived from an exploratory model rather than the primary time‐to‐event framework. Their inclusion reflects the availability of these covariates in the TriNetX dataset and prior literature suggesting potential associations with fall risk. However, because these variables were not consistently available across patients and were not central to our primary hypothesis, they were excluded from the Cox regression. We therefore present these findings as hypothesis‐generating signals that warrant further validation in prospective studies.

An additional finding was the modestly lower fall risk observed among Black patients in adjusted analyses. This result should be interpreted with caution. It is unlikely to reflect biological protection but may instead arise from differential coding practices, under‐ascertainment of falls, differences in healthcare utilization, or unmeasured social determinants of health. Prior research has shown that minority populations are often less likely to have falls documented in administrative records despite similar or higher self‐reported fall rates. Thus, this apparent protective effect may represent systematic under‐recording or other unmeasured confounding, rather than a true reduction in fall risk. Future studies that incorporate patient‐reported falls and objective performance assessments will be necessary to clarify whether this pattern reflects coding bias or real differences in risk.

The implications for clinical care are notable. Screening for sarcopenia has been recommended in geriatric populations, but our results argue that in breast cancer survivors, screening should be integrated with a comprehensive evaluation of comorbidities and functional status. Sarcopenia prevalence is high in older women with cancer, and prior research has linked it with reduced quality of life, poorer tolerance of chemotherapy, and higher healthcare utilization [23]. Detecting sarcopenia early may therefore help identify women at risk not only for falls but also for a spectrum of adverse outcomes. At the same time, interventions must be multifaceted. Exercise‐based strategies, particularly resistance and balance training, remain central to improving muscle strength and function. Nutritional support, including adequate protein intake and supplementation when appropriate, can further support muscle mass preservation. Importantly, these strategies should be coupled with careful management of hypertension, diabetes, neuropathy, and polypharmacy, as these factors substantially modify fall risk in this group.

Our disease‐specific focus on breast cancer is supported by several mechanistic and clinical considerations. Compared with other malignancies, breast cancer treatments more frequently involve long‐term endocrine therapy, which can accelerate sarcopenia through estrogen suppression [16]. Chemotherapy‐induced peripheral neuropathy, common in breast cancer, directly contributes to balance dysfunction and fall risk [13, 14, 22]. Additionally, the survivorship phase in breast cancer often involves extended periods of fatigue, pain, or musculoskeletal discomfort, further limiting activity and accelerating muscle loss [4, 5, 15]. These intersecting vulnerabilities make breast cancer survivors a high‐priority group for integrated sarcopenia screening and fall prevention strategies [9, 10].

The psychological and treatment‐related dimensions of fall risk also warrant consideration. Chemotherapy‐induced peripheral neuropathy, chronic fatigue, pain, and cognitive changes can exacerbate the impact of sarcopenia on balance and mobility [17]. Hormonal therapy and its musculoskeletal side effects may also contribute [16]. Integrating mental health support and survivorship counseling into care plans could therefore enhance resilience and adherence to physical activity interventions. Evidence from multidisciplinary programs suggests that addressing muscle strength, nutrition, and psychological well‐being together may slow, or in some cases reverse, sarcopenia‐related decline [24].

Surgical treatment may also influence sarcopenia and fall risk through its impact on postoperative mobility and recovery [25]. More extensive procedures, such as mastectomy with axillary lymph node dissection, are associated with greater physical burden, longer recovery time, and potential functional decline [26]. In contrast, newer minimally invasive techniques for non‐palpable breast cancer, including vacuum‐assisted breast biopsy (VABB) and radio‐guided occult lesion localization (ROLL), may offer reduced postoperative complications and better preservation of physical function [27]. These approaches may help mitigate treatment‐related deconditioning in older patients. Future research could explore whether the surgical approach modifies the relationship between sarcopenia, recovery, and fall outcomes.

## 5 Strengths and Limitations

A major strength of this study is the use of a large, multi‐institutional dataset that enabled the analysis of over 880,000 breast cancer patients, including a clinically meaningful subset with sarcopenia. The scale of the dataset provided statistical power to detect differences in fall risk even for relatively rare outcomes and allowed the application of advanced analytic methods such as propensity score matching and Cox proportional hazards modeling. The availability of detailed diagnostic and medication codes also permitted adjustment for a broad array of comorbidities and exposures that influence fall risk. By leveraging contemporary electronic health records, the study reflects real‐world clinical practice and enhances generalizability to diverse populations.

Several limitations should be acknowledged. First, the reliance on administrative ICD‐10 codes to define both sarcopenia (M62.84) and falls may introduce misclassification bias. Sarcopenia is underdiagnosed in routine clinical practice, and coding typically reflects more severe or symptomatic cases, leading to underestimation of true prevalence and exclusion of subclinical disease. Sarcopenia is underdiagnosed in clinical practice, and its coding frequency is low compared with its true prevalence in older adults. Prior work has shown that reliance on administrative codes tends to capture patients with more advanced or clinically evident muscle loss, while milder or subclinical cases are often missed. As a result, our operational definition may underestimate the prevalence of sarcopenia and preferentially identify more severe cases, which could bias associations toward stronger observed effects. Similarly, while our data capture included falls recorded during inpatient, outpatient, and emergency department visits, fall events that were non‐injurious, undocumented, or did not prompt clinical care are likely under‐recorded, reflecting a well‐recognized limitation of ICD‐based surveillance. Second, residual confounding is possible despite propensity score matching and multivariable adjustment, as important factors such as physical performance measures, nutritional status, and patient‐reported outcomes were unavailable in the dataset. Third, we could not evaluate medication adherence, cancer stage detail, or longitudinal changes in muscle mass, which may further influence risk. Fourth, the retrospective design precludes causal inference, and findings should be interpreted as associations rather than causal effects. Finally, although TriNetX aggregates data from multiple healthcare systems, patients who sought care outside participating institutions may not be fully represented. These limitations underscore the importance of future studies that incorporate objective measures of muscle function (e.g., grip strength, gait speed, or CT‐derived muscle indices) to more accurately characterize sarcopenia‐related fall risk in breast cancer populations. Finally, while the prevalence of sarcopenia in our study was low (0.09%), which can pose challenges for propensity score matching, we were able to match all 768 exposed patients to unexposed comparators without exclusions. Post‐matching balance was excellent across all baseline variables, with standardized mean differences < 0.02. Nonetheless, we acknowledge that matched analyses based on rare exposures should be interpreted cautiously and viewed as exploratory rather than definitive.

## 6 Conclusion

Despite these limitations, this study adds important evidence to the growing literature on survivorship and aging in breast cancer. By focusing on a high‐risk, understudied group of older women, we provide actionable data to inform clinical care and future research. Efforts to prevent falls in this population will likely require a multifaceted approach that goes beyond sarcopenia alone, addressing the full spectrum of functional, medical, and treatment‐related risk factors.

In conclusion, sarcopenia is a significant marker of increased fall risk in older breast cancer survivors, but much of this risk is shared with other age‐related and clinical factors. Integrating sarcopenia assessment into routine survivorship care, while simultaneously managing comorbidities and supporting physical function, offers a promising pathway to improve safety and quality of life for this growing population.

## Author Contributions

**Asmaa Namoos:** conceptualization, methodology, investigation, validation, software, data curation, visualization, writing – original draft, writing – review and editing, formal analysis. **Rana Ramadan:** writing – original draft, writing – review and editing, methodology. **Dina Ramadan:** methodology, writing – review and editing. **Annie Liang:** writing – review and editing. **Nicholas Thomson:** conceptualization, investigation, funding acquisition, writing – original draft, writing – review and editing, visualization, validation, methodology, project administration; resources.

## Ethics Statement

This study was conducted using deidentified data from the TriNetX platform. Institutional review board approval was not required per federal regulations on secondary analysis of anonymized data.

## Conflicts of Interest

The authors declare no conflicts of interest.

## Data Availability

The data supporting the findings of this study are available from TriNetX but restrictions apply to the availability of these data, which were used under license for the current study and are not publicly available.

## References

[1] A. A. Z. Abu Bakar , A. Abdul Kadir , N. S. Idris , and S. N. Mohd Nawi , “Older Adults With Hypertension: Prevalence of Falls and Their Associated Factors,” International Journal of Environmental Research and Public Health 18, no. 16 (2021): 8257. 34444005 10.3390/ijerph18168257 PMC8392439

[2] G. C. Ang , S. L. Low , and C. H. How , “Approach to Falls Among the Elderly in the Community,” Singapore Medical Journal 61, no. 3 (2020): 116–121. 32488276 10.11622/smedj.2020029 PMC7905119

[3] M. K. Appeadu and B. Bordoni , “Falls and Fall Prevention in Older Adults,” in StatPearls [Internet] (StatPearls Publishing, 2024), http://www.ncbi.nlm.nih.gov/books/NBK560761/. 32809596

[4] R. King , L. Stafford , P. Butow , S. Giunta , and R. Laidsaar‐Powell , “Psychosocial Experiences of Breast Cancer Survivors: A Meta‐Review,” Journal of Cancer Survivorship 18, no. 1 (2024): 84–123. 36854799 10.1007/s11764-023-01336-x PMC10866753

[5] C. García‐Chico , S. López‐Ortiz , S. Peñín‐Grandes , et al., “Physical Exercise and the Hallmarks of Breast Cancer: A Narrative Review,” Cancers 15, no. 1 (2023): 324. 36612320 10.3390/cancers15010324 PMC9818971

[6] A. D. Ardeljan and R. Hurezeanu , “Sarcopenia,” in StatPearls [Internet] (StatPearls Publishing, 2025), http://www.ncbi.nlm.nih.gov/books/NBK560813/. 32809648

[7] W. K. Mitchell , P. J. Atherton , J. Williams , M. Larvin , J. N. Lund , and M. Narici , “Sarcopenia, Dynapenia, and the Impact of Advancing Age on Human Skeletal Muscle Size and Strength; a Quantitative Review,” Frontiers in Physiology [Internet] 3 (2012): 1–18, https://www.frontiersin.org/journals/physiology/articles/10.3389/fphys.2012.00260/full. 22934016 10.3389/fphys.2012.00260 PMC3429036

[8] V. E. Baracos and L. Arribas , “Sarcopenic Obesity: Hidden Muscle Wasting and Its Impact for Survival and Complications of Cancer Therapy,” Annals of Oncology 29, no. 1 (2018): ii1–ii9. 10.1093/annonc/mdx810 29506228

[9] M. K. Jang , S. Park , C. Park , R. Raszewski , S. Park , and S. Kim , “Sarcopenia in Patients With Metastatic Breast Cancer: A Systematic Review and Meta‐Analysis,” Breast 82 (2025): 104508. 40446428 10.1016/j.breast.2025.104508 PMC12159878

[10] V. F. de Souza Mame , R. d. A. M. Bernabé , T. G. Santos , et al., “Risk of Sarcopenia in Women With Breast Cancer: A Comparative Analysis of Screening Tools,” BMC Cancer 25, no. 1 (2025): 839. 40335951 10.1186/s12885-025-14062-7 PMC12057273

[11] Q. Li , N. Shang , Q. Gao , S. Guo , and T. Yang , “Prevalence of Sarcopenia and Its Association With Frailty and Malnutrition Among Older Patients With Sepsis‐a Cross‐Sectional Study in the Emergency Department,” BMC Geriatrics 25 (2025): 377. 40426064 10.1186/s12877-025-06060-y PMC12107726

[12] G. R. Williams , Y. Chen , K. M. Kenzik , et al., “Assessment of Sarcopenia Measures, Survival, and Disability in Older Adults Before and After Diagnosis With Cancer,” JAMA Network Open 3, no. 5 (2020): e204783. 32396194 10.1001/jamanetworkopen.2020.4783 PMC7218493

[13] K. M. Sturgeon , K. M. Mathis , C. J. Rogers , K. H. Schmitz , and D. L. Waning , “Cancer‐ and Chemotherapy‐Induced Musculoskeletal Degradation,” JBMR Plus 3, no. 3 (2019): e10187. 30918923 10.1002/jbm4.10187 PMC6419610

[14] H. Komatsu , K. Yagasaki , Y. Komatsu , et al., “Falls and Functional Impairments in Breast Cancer Patients With Chemotherapy‐Induced Peripheral Neuropathy,” Asia‐Pacific Journal of Oncology Nursing 6, no. 3 (2019): 253–260. 31259221 10.4103/apjon.apjon_7_19 PMC6518979

[15] B. I. Bodai and P. Tuso , “Breast Cancer Survivorship: A Comprehensive Review of Long‐Term Medical Issues and Lifestyle Recommendations,” Permanente Journal 19, no. 2 (2015): 48–79. 10.7812/TPP/14-241 PMC4403581 25902343

[16] S. W. Kim and R. Kim , “The Association Between Hormone Therapy and Sarcopenia in Postmenopausal Women: The Korea National Health and Nutrition Examination Survey, 2008‐2011,” Menopause (New York, N.Y.) 27, no. 5 (2020): 506–511. 32049925 10.1097/GME.0000000000001509 PMC7188056

[17] E. S. Hile , G. K. Fitzgerald , and S. A. Studenski , “Persistent Mobility Disability After Neurotoxic Chemotherapy,” Physical Therapy 90, no. 11 (2010): 1649–1657. 20813818 10.2522/ptj.20090405 PMC2967709

[18] TriNetX , “TriNetX [Internet],” (2013), https://live.trinetx.com/tnx/study/202800/analytics/new.

[19] Publication Guidelines [Internet]. TriNetX, https://trinetx.com/real‐world‐resources/case‐studies‐publications/trinetx‐publication‐guidelines/.

[20] S. S. Y. Yeung , E. M. Reijnierse , V. K. Pham , et al., “Sarcopenia and Its Association With Falls and Fractures in Older Adults: A Systematic Review and Meta‐Analysis,” Journal of Cachexia, Sarcopenia and Muscle 10, no. 3 (2019): 485–500. 30993881 10.1002/jcsm.12411 PMC6596401

[21] D. Wilson , T. Jackson , E. Sapey , and J. M. Lord , “Frailty and Sarcopenia: The Potential Role of an Aged Immune System,” Ageing Research Reviews 36 (2017): 1–10. 28223244 10.1016/j.arr.2017.01.006

[22] F. C. Martin and A. H. Ranhoff , “Frailty and Sarcopenia,” in Orthogeriatrics: The Management of Older Patients With Fragility Fractures [Internet], 2nd ed., ed. P. Falaschi and D. Marsh (Springer, 2021), http://www.ncbi.nlm.nih.gov/books/NBK565582/. 33347100

[23] M. Anjanappa , M. Corden , A. Green , et al., “Sarcopenia in Cancer: Risking More Than Muscle Loss,” Technical Innovations & Patient Support in Radiation Oncology 16 (2020): 50–57. 33385074 10.1016/j.tipsro.2020.10.001 PMC7769854

[24] W. T. Park , O. J. Shon , and G. B. Kim , “Multidisciplinary Approach to Sarcopenia: A Narrative Review,” Journal of Yeungnam Medical Science 40, no. 4 (2023): 352–363. 37674374 10.12701/jyms.2023.00724 PMC10626311

[25] K. Chang , J. A. Albright , E. J. Testa , A. B. Balboni , A. H. Daniels , and E. Cohen , “Sarcopenia Is Associated With an Increased Risk of Postoperative Complications Following Total Hip Arthroplasty for Osteoarthritis,” Biology 12, no. 2 (2023): 295. 36829571 10.3390/biology12020295 PMC9953618

[26] N. A. Che Bakri , R. M. Kwasnicki , N. Khan , et al., “Impact of Axillary Lymph Node Dissection and Sentinel Lymph Node Biopsy on Upper Limb Morbidity in Breast Cancer Patients,” Annals of Surgery 277, no. 4 (2023): 572–580. 35946806 10.1097/SLA.0000000000005671 PMC9994843

[27] K. Ocal , A. Dag , O. Turkmenoglu , E. C. Gunay , E. Yucel , and M. N. Duce , “Radioguided Occult Lesion Localization Versus Wire‐Guided Localization for Non‐Palpable Breast Lesions: Randomized Controlled Trial,” Clinics 66, no. 6 (2011): 1003–1007. 21808866 10.1590/S1807-59322011000600014 PMC3129952

